# Minimal genetic differentiation of the malaria vector *Nyssorhynchus darlingi* associated with forest cover level in Amazonian Brazil

**DOI:** 10.1371/journal.pone.0225005

**Published:** 2019-11-14

**Authors:** Catharine Prussing, Kevin J. Emerson, Sara A. Bickersmith, Maria Anice Mureb Sallum, Jan E. Conn

**Affiliations:** 1 University at Albany, State University of New York, School of Public Health, Department of Biomedical Sciences, Albany, NY, United States of America; 2 Department of Biology, St. Mary’s College of Maryland, St. Mary’s City, MD, United States of America; 3 Wadsworth Center, New York State Department of Health, Albany, NY, United States of America; 4 Departamento de Epidemiologia, Faculdade de Saúde Pública, Universidade de São Paulo, São Paulo, SP, Brasil; United States Department of Agriculture, UNITED STATES

## Abstract

The relationship between deforestation and malaria in Amazonian Brazil is complex, and a deeper understanding of this relationship is required to inform effective control measures in this region. Here, we are particularly interested in characterizing the impact of land use and land cover change on the genetics of the major regional vector of malaria, *Nyssorhynchus darlingi* (Root). We used nextera-tagmented, Reductively Amplified DNA (nextRAD) genotyping-by-sequencing to genotype 164 *Ny*. *darlingi* collected from 16 collection sites with divergent forest cover levels in seven municipalities in four municipality groups that span the state of Amazonas in northwestern Amazonian Brazil: São Gabriel da Cachoeira, Presidente Figueiredo, four municipalities in the area around Cruzeiro do Sul, and Lábrea. Using a dataset of 5,561 Single Nucleotide Polymorphisms (SNPs), we investigated the genetic structure of these *Ny*. *darlingi* populations with a combination of model- and non-model-based analyses. We identified weak to moderate genetic differentiation among the four municipality groups. There was no evidence for microgeographic genetic structure of *Ny*. *darlingi* among forest cover levels within the municipality groups, indicating that there may be gene flow across areas of these municipalities with different degrees of deforestation. Additionally, we conducted an environmental association analysis using two outlier detection methods to determine whether individual SNPs were associated with forest cover level without affecting overall population genetic structure. We identified 14 outlier SNPs, and investigated functions associated with their proximal genes, which could be further characterized in future studies.

## Introduction

In the Amazon region, there is an increasing understanding that land use and land cover (LULC) changes caused by agricultural activity, logging, and road construction are modifying the risk of human malaria infection [1–6]. In Amazonian Brazil, malaria rates have been shown to increase during the earliest stages of settlement, when rainforest is first being cleared, humans are settling near the forest fringe, and immunity is low [7, 8]. Though a direct, positive relationship between deforestation and malaria rates in the Amazon has been reported in numerous studies [1, 2, 5, 6], other studies have found the opposite result [4, 9]. These discrepancies could stem from inconsistencies in defining forest cover and deforestation, and highlight the complex relationship between landscape change and malaria risk [4]. Furthermore, it is possible that such discrepancies stem from differences in the effects of landscape changes on the vectorial capacity of genetically distinct mosquito populations [10].

In the Amazon, the major vector of malaria is *Nyssorhynchus darlingi* (Root, also known as *Anopheles darlingi* Root; we have followed the recommendation in [11] to elevate the subgenus *Nyssorhynchus* to a genus). Deforested areas have been associated with an increased *Ny*. *darlingi* human biting rate [12, 13] and increased larval habitat suitability for *Ny*. *darlingi* [14–16]. LULC changes have also been associated with changes in mosquito species composition, with *Ny*. *darlingi* abundance generally highest in human-modified and deforested landscapes [17–20]. Deforestation might additionally impact the population genetic structure of malaria vectors. Genetic differentiation between and within members of *Anopheles gambiae* Giles s.l. has been associated with agricultural activity and the degree of urban/built environment landscapes [21, 22]. Similarly, population structure was detected between *Ny*. *darlingi* collected from two Brazilian settlements separated by 60 km with very different forest cover levels [23]. The possibility of microgeographic genetic differentiation of *Ny*. *darlingi* among areas with different forest covers has implications for malaria transmission, as subpopulations of *Ny*. *darlingi* may differ in vector competence or vectorial capacity, or in their response to vector control activities. Differences in *Plasmodium* infection rates have previously been identified across genetically distinct populations of Anophelinae species [24–26].

Brazil accounts for ~25% of malaria cases reported from the Americas, and has reported 100,000–200,000 cases annually since 2013 [27]. Over 99% of the malaria burden in Brazil is concentrated in the Amazon Basin, particularly in the states of Amazonas and Acre [28, 29]. Though the majority of malaria cases in these states, as in all of Brazil, are caused by *Plasmodium vivax*, these states also have a persistently and disproportionately high proportion of cases caused by *Plasmodium falciparum*, which is associated with higher morbidity and mortality [29]. Within these states, malaria transmission is heterogeneous [30, 31], with pockets of high malaria infection rates in human and mosquito populations [32].

In Brazil, nextera-tagmented Reductively Amplified DNA (nextRAD) genotyping-by-sequencing detected genetic clustering of *Ny*. *darlingi* into three groups by biogeographical region (southeast, west Atlantic, and Amazon) [33]. These results are consistent with previous studies based on microsatellites [34, 35] and *COI* sequencing [36] that have shown little genetic differentiation of *Ny*. *darlingi* across Western and Central Amazonian Brazil. At the microgeographic level, low levels of genetic differentiation have been detected between *Ny*. *darlingi* collected in different seasons [37] and habitats [13] using microsatellites. Despite evidence that deforestation may impact the population genetic structure of *Ny*. *darlingi* [23], this question has not been investigated at a broader geographic scale. To address this, we used nextRAD genotyping-by-sequencing to determine whether forest cover level is associated with microgeographic genetic structuring of *Ny*. *darlingi* in four municipality groups in Amazonas and Acre states in Amazonian Brazil.

## Methods

### Study site selection and adult *Ny*. *darlingi* collections

**Fig 1:**
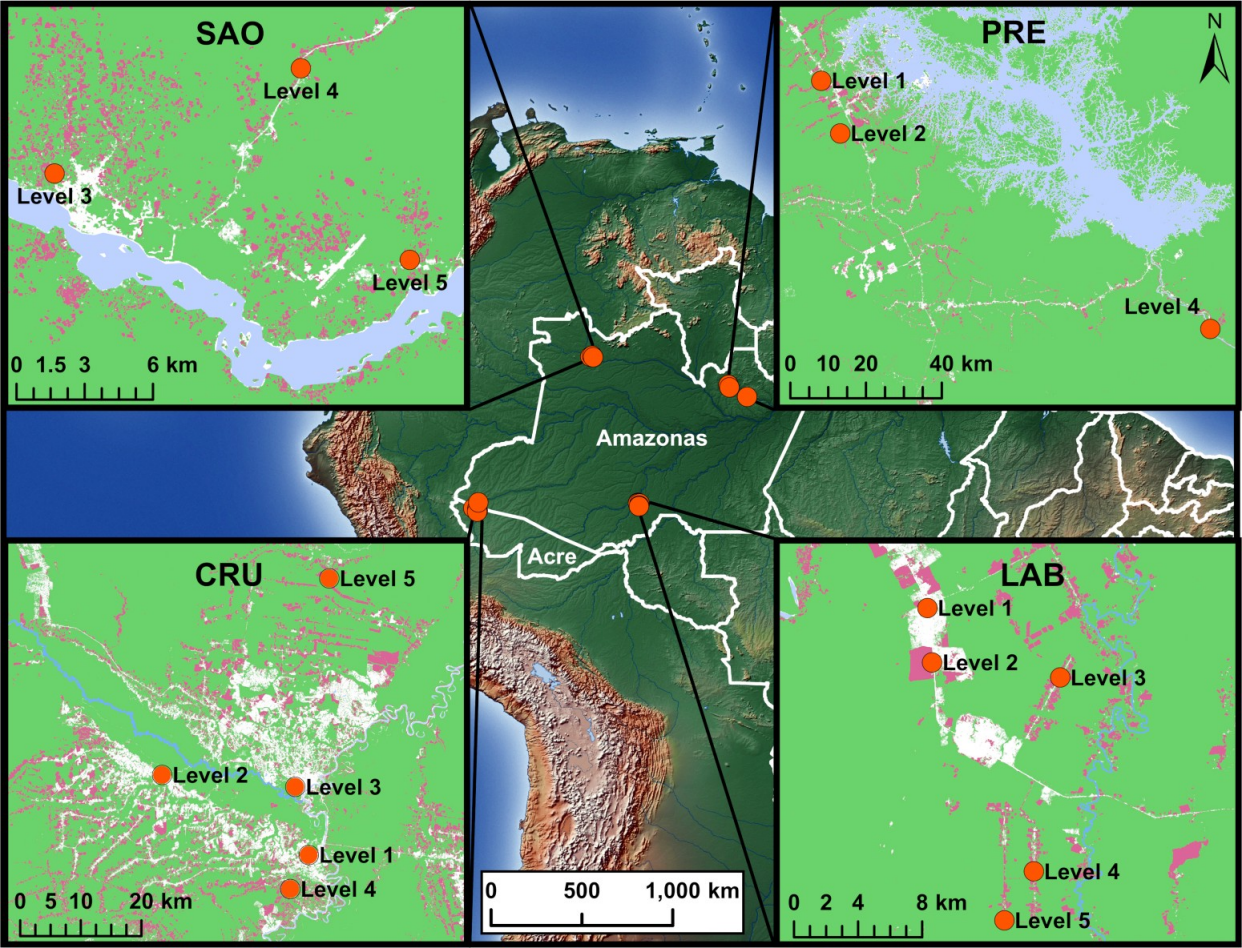
Map of *Ny*. *darlingi* collection sites in Amazonas and Acre States, Brazil, with insets for each municipality group: SAO: São Gabriel da Cachoeira, PRE: Presidente Figueiredo, CRU: Cruzeiro do Sul area, LAB: Lábrea. Each collection site within each municipality group is indicated by an orange dot, with Levels 1–5 in the municipality insets indicating the forest cover level (Level 1 = ~0–20% forest cover in a 1km radius, Level 2 = ~20–40% forest cover, etc.). Green color on inset map indicates areas of tree canopy cover as of the year 2000, pink color indicates forest loss during the period 2000–2014 (Hansen/UMD/Google/USGS/NASA, http://data.globalforestwatch.org/); and blue color indicates bodies of water (ESRI World Water Bodies/World Linear Water layers). Basemap: Natural Earth (https://www.naturalearthdata.com/).

Adult female *Ny*. *darlingi* were collected in 2015–2017 from 16 sites spread across seven municipalities in Amazonas and Acre States (Fig 1). For the purposes of this study, the geographically proximal municipalities of Cruzeiro do Sul, Rodrigues Alves, Mâncio Lima, and Guajará were grouped together as one municipality group (Cruzeiro do Sul area), and Lábrea, Presidente Figueiredo, and São Gabriel da Cachoeira were each considered a separate municipality group, for a total of four municipality groups. The collection methods for this study are described in detail in [32]. Briefly, houses at least 2.5km apart were selected as collection sites within each municipality. Mosquitoes were collected by human landing catch (HLC) in the peridomestic area, within ~5m of each house. Collections were done from 6pm to 12am during the dry season and wet-dry season transition (between April and November). Mosquitoes were euthanized with ethyl acetate in the field, stored on silica gel, and identified morphologically to species using entomological keys [38].

All necessary permits were obtained for the field collections. Collections were made under permanent permit number 12583–1 from Instituto Chico Mendes de Conservação da Biodiversidade (ICMBio, SISBIO). Specific permission was not required for these locations as permission to collect was granted under the permanent permit. The collection locations were not privately owned or protected, and the field studies did not involve protected or endangered species.

For each collection site, forest cover in a 1 km radius around the site was calculated; this radius was selected to reflect the approximate maximum flight range of *Ny*. *darlingi* [39]. Forest cover was calculated using European Space Agency (ESA) Sentinel 2A satellite imagery from the closest possible date to the field collection, minimizing cloud coverage. Radiometric and atmospheric corrections were performed using the ESA’s Sen2Cor software v2.4.0 [40]. Maximum likelihood supervised classification was used to assign each pixel as forest or non-forest using spectral bands 2, 3, and 4. The percentage forest cover was calculated for each collection site using the formula $\%\;Forest\;Cover=\frac{\sum_{j=1}^{n}a_{ij}}{A}(100)$, where a_ij_ corresponds to the forest area and A corresponds to the total area of the landscape.

For each municipality group, collection sites where at least 10 *Ny*. *darlingi* were collected were selected to cover the maximum possible range of forest cover percentage within each municipality group. Collection sites were split approximately into quintiles by forest cover percentage (level 1 = ~0–20% forest cover, level 2 = ~20–40% forest cover etc.). *Nyssorhynchus darlingi* were available from all 5 forest cover levels for two municipality groups (Lábrea and Cruzeiro do Sul-area), and from 3 levels for the remaining two municipality groups (Presidente Figueiredo and São Gabriel da Cachoeira), for a total of 16 collection sites across the four municipality groups. Genomic DNA was extracted from individual *Ny*. *darlingi* using the Qiagen DNeasy Blood & Tissue Kit (Hilden, Germany), and DNA concentrations measured using a Qubit Fluorometer (Life Technologies, Carlsbad, CA, USA). For genotyping, 15 *Ny*. *darlingi* with DNA concentration ≥0.5 ng/μL were selected from each level 1 and level 5 collection site, and 10 from each level 2, 3, and 4 site, for a total of 190 *Ny*. *darlingi* (Table 1).

**Table 1:** Forest cover, collection date, and sample size for the 16 collection sites.

Municipality	Forest Cover Level	% Forest Cover in 1km radius	Collection Date	# *Ny*. *darlingi* genotyped	# *Ny*. *darlingi* in final analysis^2^
**Rodrigues Alves^1^**	1	24%	6/30/2017	15	12
**Mâncio Lima^1^**	2	43%	5/28/2015	10	9
**Cruzeiro do Sul^1^**	3	58%	4/19/2015	10	7
**Rodrigues Alves^1^**	4	70%	6/26/2017	10	9
**Guajará^1^**	5	83%	7/14/2017	15	14
**Lábrea**	1	19%	8/9/2015	15	13
**Lábrea**	2	31%	8/10/2015	10	9
**Lábrea**	3	59%	8/2/2015	10	10
**Lábrea**	4	77%	8/5/2015	10	10
**Lábrea**	5	87%	8/4/2015	15	14
**Presidente Figueiredo**	1	18%	8/20/2017	15	12
**Presidente Figueiredo**	2	30%	8/17/2017	10	8
**Presidente Figueiredo**	4	72%	8/19/2017	10	6
**São Gabriel da Cachoeira**	3	40%	11/13/2017	10	9
**São Gabriel da Cachoeira**	4	69%	11/9/2017	10	9
**São Gabriel da Cachoeira**	5	78%	11/18/2017	15	13
**Total**				190	164

^1^Cruzeiro do Sul area

^2^Includes only individuals matching to at least 10,000 catalog loci during STACKS analysis.

### nextRAD genotyping-by-sequencing

Genomic DNA from the 190 individuals was converted into nextRAD genotyping-by-sequencing libraries by SNPsaurus, LLC as in [41]. Genomic DNA was first fragmented with Nextera reagent (Illumina, Inc., San Diego, CA, USA), which also ligates short adapter sequences to the ends of the fragments. The Nextera reaction was scaled down to fragment 3 ng of genomic DNA (the kit is optimized to fragment 50 ng). Fragmented DNA was then amplified using the Phusion Hot Start Flex DNA Polymerase (New England Biolabs, Inc., Ipswich, MA) for 25 cycles at 75°C, with one of the primers matching the adapter and extending 8 nucleotides into the genomic DNA with the selective sequence TGCAGGAG. Thus, only fragments starting with a sequence that can be hybridized by the selective sequence of the primer will be efficiently amplified. The nextRAD libraries were sequenced on a HiSeq 4000 with two lanes of 150 bp reads (University of Oregon). All sequencing reads were uploaded to the NCBI SRA database (BioProject ID: PRJNA545461).

### Sequence processing

Raw sequence reads were analyzed using STACKS v2.3b [42, 43]. Briefly, the STACKS pipeline collects raw sequencing reads together into matching stacks, then builds a catalog of putative consensus RAD loci, which span the length of the amplified RAD fragments, by combining stacks from multiple individuals. The pipeline then matches individuals against the catalog of loci, and calls SNPs for each individual at each locus based on a maximum likelihood framework. Finally, the set of loci is filtered based on their frequencies in the study populations. Low-quality reads were dropped using the STACKS *process_radtags* program, and *ustacks* was used to align reads into stacks, with the minimum depth of coverage required to create a stack set to 3, the maximum distance allowed between stacks set to 4, and the maximum distance allowed to align secondary reads to primary stacks set to 6. A catalog of putative loci was built using 24 representative *Ny*. *darlingi* individuals (S3 File) collected from Brazil and Peru, including 5 individuals from the current study, 4 from [44], 2 from [33], and the remainder from Chu *et al*. (manuscript submitted). All 24 individuals were sequenced, and the reads processed in *ustacks*, using the methods described above. The catalog was built using the STACKS *cstacks* program, allowing 4 mismatches between stacks, with gapped alignments enabled. The catalog loci were mapped to the *Ny*. *darlingi* genome (AdarC3) [45] using BWA MEM with default parameters [46]; only loci mapping to the genome were retained. After processing in *ustacks*, stacks from the 190 individuals in the current study were searched against the catalog, and SNPs called, with the STACKS *sstacks*, *tsv2bam*, and *gstacks* programs using default settings.

To control for quality and sequence coverage variation among individuals, only individuals that matched to at least 10,000 catalog loci were included in the analysis (n = 164, including at least 6 individuals from each municipality group/deforestation level combination; Table 1). The STACKS *populations* program was used to select the first SNP from each RAD locus found in all 4 municipality groups, and in at least 50% of the individuals in each municipality group, with the minimum minor allele frequency set to 0.02 and the maximum observed heterozygosity set to 0.7. A bash script including the STACKS parameters used is included as S4 File, and the STRUCTURE file used for subsequent analyses is included as S5 File.

### Population structure analysis

STRUCTURE v2.3.4 [47] was run using the program StrAuto [48] for 10 replicates each of *K* = 1 to 8, with a burn-in of 100,000 generations and an MCMC chain of 1,000,000 generations. The Evanno method [49] implemented in STRUCTURE Harvester [50] was used to determine the optimal number of genetic clusters. CLUMPP v1.1.2 [51] was run using default settings, and STRUCTURE plots visualized, using the R v3.5.2 [52] package *pophelper* v2.2.7 [53]. As a less computationally intensive method to investigate substructure within municipality groups, fastStructure [54] analysis was run for the full dataset (to confirm that results were comparable to STRUCTURE results) and then for each municipality group separately using default settings for five replicates each of *K* = 1 to 10. The replicate runs were combined using CLUMPP and visualized in *pophelper* as above.

Principal components analysis (PCA) was performed using the R package *ade4* v1.7–13 [55] dudi.pca() function, and PCA plots were created using the R package *factoextra* v1.0.5 [56] fviz_pca_ind() function. Discriminant Analysis of Principal Components (DAPC) [57] was performed using the R package *adegenet* v2.1.1 [58].

A hierarchical Analysis of Molecular Variance (AMOVA), with individuals grouped into forest cover levels within municipality groups, was calculated using the poppr.amova() function in the R package *poppr* v2.8.2 [59]. Pairwise *F*_*ST*_ values with confidence intervals using 999 bootstrap samples were calculated using the stamppFst() function in the R package *StAMPP* v1.5.1 [60]. Isolation by distance was examined by plotting the geographic distance between each collection site vs. Prevosti’s genetic distance (calculated using the dist.genpop() function in *adegenet*) and calculating a Mantel test to compare the two distance matrices using the mantel.randtest() function in *ade4*.

### Outlier analysis

Two methods were used to identify SNPs associated with forest cover. First, latent-factor mixed modelling (LFMM) [61] was run using the R package *LEA* v2.4.0 [62]. In preparation for LFMM, a sparse non-negative matrix factorization (sNMF) [63] analysis completed using *LEA* was run with ten repetitions of *K* = 1 through 10. The optimal number of populations, where the cross-entropy curve was at a minimum, was four. The sNMF results were used to impute missing genotypes using the *LEA* impute() function. Five repetitions of LFMM were run on the imputed dataset, with a burn-in of 5000 and 10000 iterations for each, adjusting for four latent factors (as suggested by optimal number of populations from the sNMF analysis), with forest cover percentage as the explanatory variable. The z-scores from the five runs were combined, and the *p*-values adjusted for multiple testing using the Benjamini-Hochberg procedure as recommended by the authors [61]. SNPs with an adjusted *p*-value of 0.05 were considered outliers.

As a second method to identify SNPs associated with forest cover, bayenv2 [64] was run, following conversion of the vcf file in PGDSpider v2.1.1.5 [65], with each collection site as a population. Three replicate covariance matrices were computed, using 100,000 iterations. As no significant differences between the three matrices were detected using the cortest() function in the R package *psych* v1.8.12 [66], the first matrix was used. bayenv2 was run using the correlation matrix and the standardized forest cover percentage for each population, for 100,000 iterations, using the -c flag to include non-parametric tests. SNPs that were within both the top 5% of Bayes factors and the top 10% of Spearman’s *ρ* values were considered outliers. The final set of outlier SNPs includes only SNPs identified using both LFMM and bayenv2.

### Gene ontology analysis

Genes in the annotated *Ny*. *darlingi* genome scaffolds [67] located within 100 kb of each outlier SNP were investigated for gene function. A wide search window was selected because this was intended to be a broad, exploratory investigation with the goal of hypothesis generation. *Drosophila* orthologs of all genes were investigated. In addition, a gene ontology (GO) enrichment analysis was performed using the R package *topGO* v2.34.0 [68] to determine whether particular GO terms were enriched among these genes compared to the rest of the genome. Separate Fisher tests were calculated for each sub-ontology (BP: biological process, CC: cellular component, MF: molecular function) using the weight01 algorithm and a cut-off *p*-value of 0.01.

## Results

An average of 3,191,681 quality-filtered reads were sequenced from each of the 190 individual *Ny*. *darlingi* (range 359,366–7,845,737). From these, an average of 30,025 stacks per individual (range 259–409,973) made up of 643,458 total reads (range 2,035–6,430,931) matched to the catalog (S2 File). The final dataset includes one biallelic SNP from each of 5,561 loci from 164 individuals meeting the filtering constraints described in the Methods (S5 File). The average sequencing depth across all loci and individuals was 35X (range per locus: 7X-150X, SD 16X; per individual: 8X-97X, SD 19X).

### Population structure

**Fig 2:**
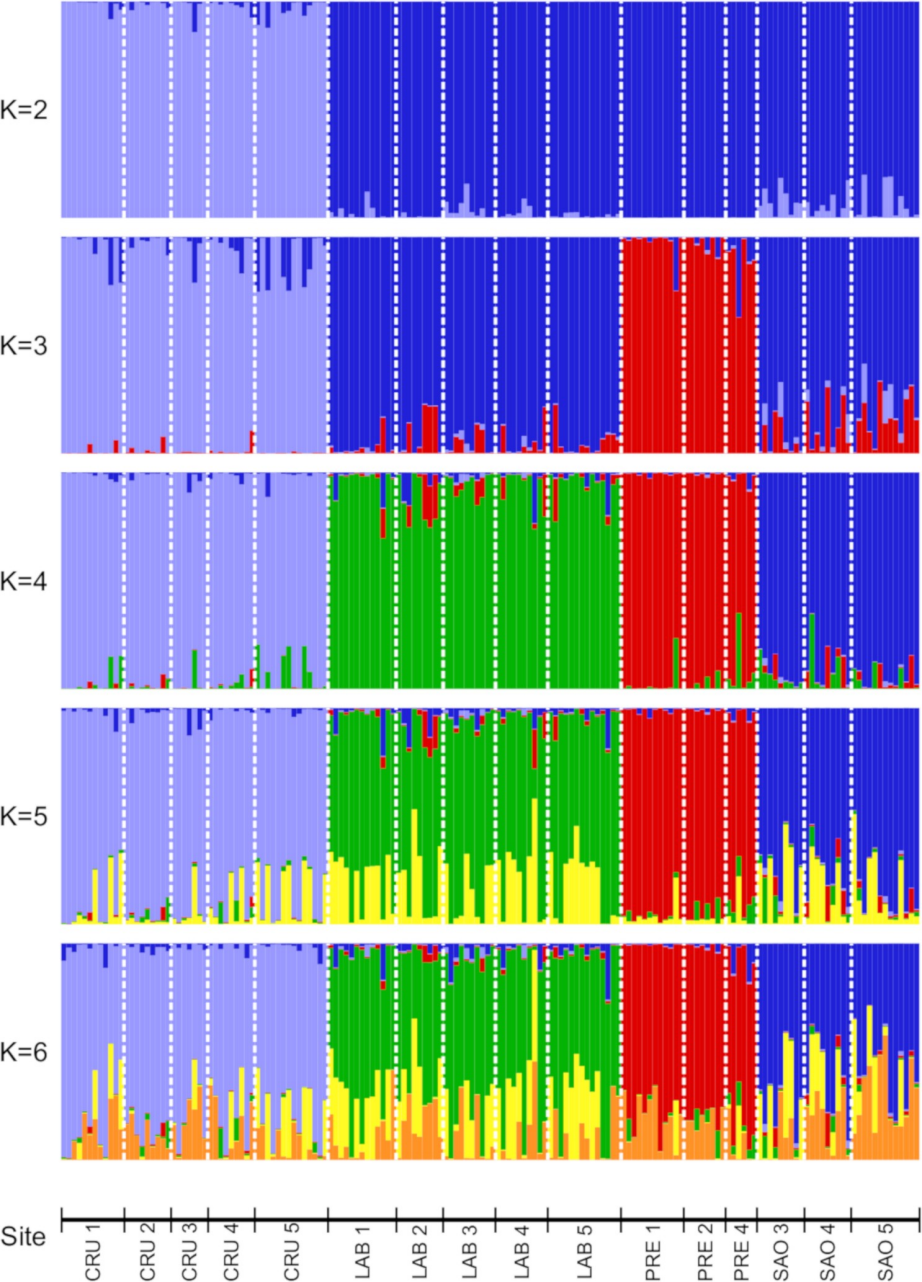
Results of STRUCTURE analysis of the 5,561 SNP dataset, comparing *Ny*. *darlingi* collected from different municipality groups (CRU: Cruzeiro do Sul area, LAB: Lábrea, PRE: Presidente Figueiredo, SAO: São Gabriel da Cachoeira) and forest cover levels (1–5), depicting *K* = 2–6 inferred clusters.

STRUCTURE Harvester analysis determined that the optimal number of genetic clusters was two, though the estimated natural log probability of the data (lnPr(X|K)) started leveling off at *K* = 5 (Fig A in S1 File). STRUCTURE results for *K* = 2–6 are shown in Fig 2, with individuals grouped by municipality group and forest cover level. Overall, there is evidence for structure among the four municipality groups, particularly between the Cruzeiro do Sul area and the other three municipalities, but no evidence of substructure among forest cover levels within each municipality group. fastStructure results were consistent with the STRUCTURE results (Fig B Panel A in S1 File), and separate fastStructure analyses for each municipality group confirmed that there was no evidence for microgeographic structure within each municipality group (Fig B Panels B-E in S1 File).

**Fig 3:**
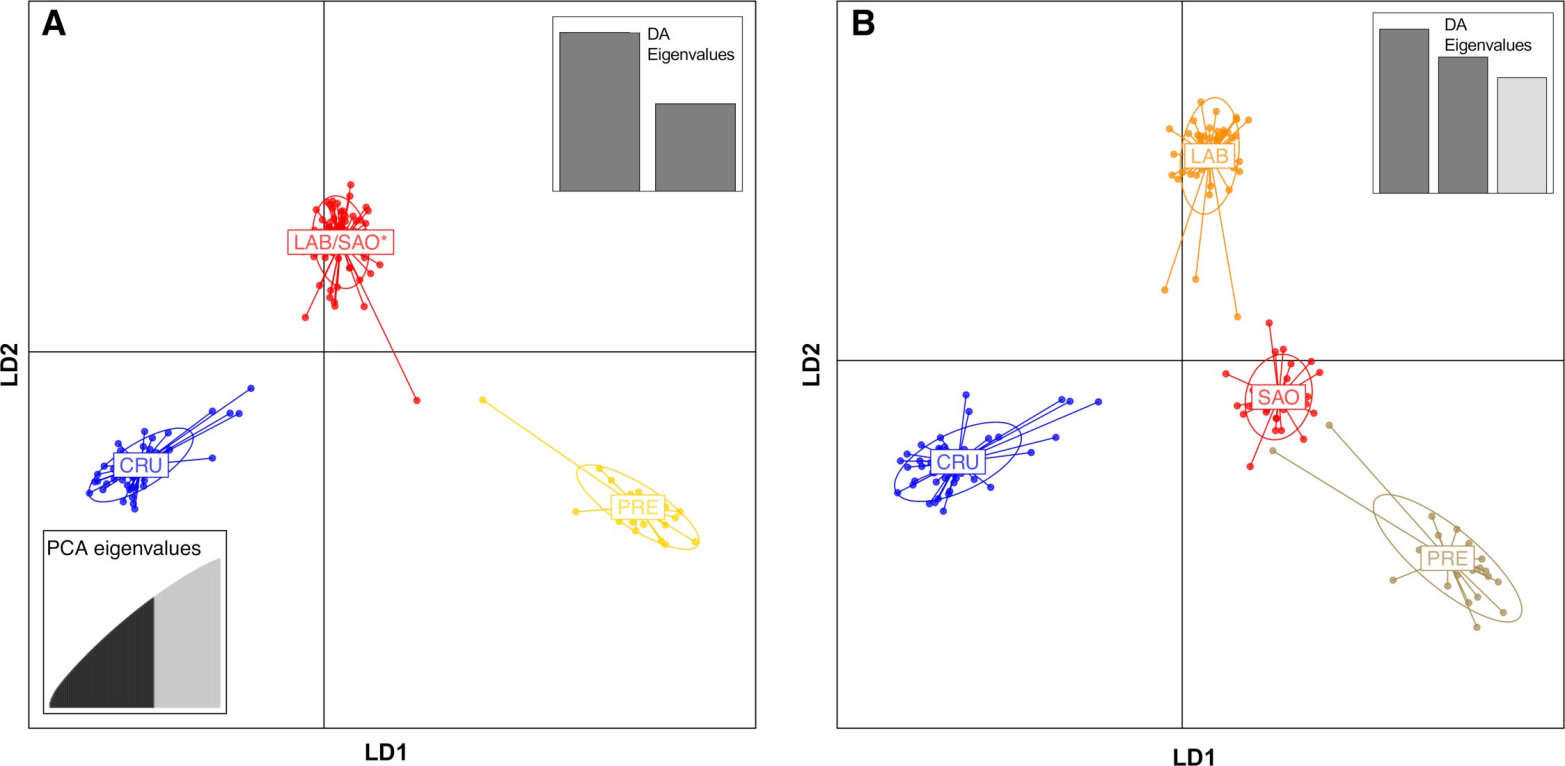
Results of Discriminant Analysis of Principal Components (DAPC) using the 5,561 SNP dataset. Ordination plots for DAPCs of three (A) and four (B) clusters, with insets showing the distribution of eigenvalues for the principal components analysis (PCA) and discriminant analysis (DA). For both analyses, 100 principal components were retained in the PCA, accounting for 73.8% of the variance in the dataset. CRU: Cruzeiro do Sul area, LAB: Lábrea, PRE: Presidente Figueiredo, SAO: São Gabriel da Cachoeira. *SAO/LAB cluster in (A) also includes one individual from PRE.

A PCA similarly showed separation among CRU, PRE, and LAB/SAO along the first two dimensions, and between LAB and SAO along the third dimension (Fig C in S1 File). Though the Bayesian Information Criterion (BIC) of the *k*-means clustering algorithm used in preparation for DAPC indicated that the optimal number of clusters was one, the Akaike Information Criterion (AIC) indicated that the optimal number of clusters was three or four (Fig D in S1 File). Setting the number of clusters to four discriminated perfectly among the four municipality groups, while setting the number of clusters to three collapsed SAO, LAB, and one individual from PRE into a single cluster (Fig 3).

By hierarchical AMOVA (Table 2), 95% of the total genetic variation was within or between individuals, 4.5% was between municipality groups, and only 0.5% was among forest cover levels within municipality groups, supporting weak genetic differentiation between the municipality groups and very little differentiation among forest cover levels. Pairwise *F*_*ST*_ values between municipality groups ranged from 0.026 (Lábrea vs. São Gabriel) to 0.075 (Presidente Figueiredo vs. Cruzeiro do Sul area) (Table 3). There was evidence for isolation by distance (Mantel *r* = 0.84, *p* = 0.001, Fig E in S1 File).

**Table 2:** Analysis of molecular variance (AMOVA), with individual *Ny*. *darlingi* nested within forest covers nested within municipality groups.

Source of variation	Degrees of freedom	Sum of squares	Variance components	Percentage of variation	*p*-value*
Among municipality groups	3	17634.98	54.83	4.54	0.001
Among forest cover levels within municipality groups	12	18259.70	6.54	0.54	0.001
Among individuals within forest cover levels	148	205587.54	242.10	20.03	0.001
Within individuals	164	148406.03	904.91	74.89	0.001
Total	327	389888.25	1208.38	100.00	

*based on 999 Monte Carlo permutation tests

**Table 3:** Pairwise *F*_*ST*_ values between municipality groups (CRU: Cruzeiro do Sul area, LAB: Lábrea, PRE: Presidente Figueiredo, SAO: São Gabriel da Cachoeira) with 95% confidence intervals based on 999 bootstrap samples (below the diagonal), and geographic distances between the centroid of collection sites in each municipality group (above the diagonal).

	CRU	LAB	PRE	SAO
**CRU**	-	884 km	1558 km	1044 km
**LAB**	0.042 (0.038, 0.045)	-	820 km	849 km
**PRE**	0.075 (0.071, 0.079)	0.052 (0.048, 0.055)	-	808 km
**SAO**	0.043 (0.040, 0.046)	0.026 (0.024, 0.028)	0.055 (0.051, 0.059)	-

### Outlier and gene ontology analyses

To determine whether there were individual SNPs associated with forest cover percentage, we conducted environmental association analyses using the 5,561 SNP dataset. LFMM detected 67 SNPs associated with forest cover percentage, and bayenv2 detected 139. Of these, 14 SNPs were detected by both methods (Figs F-G in S1 File). In the *Ny*. *darlingi* genome scaffolds, 150 genes are located within 100 kb of these 14 SNPs. These genes have a variety of functions in numerous structural and cell signaling pathways (S6 File). One (ADAC006142) encodes a venom allergen. The topGO analysis found six GO terms significantly enriched among these 150 genes compared to the rest of the *Ny*. *darlingi* genome (Table A in S1 File): GO:0035278 (miRNA mediated inhibition of translation), GO:0018149 (peptide cross-linking), GO:0005576 (extracellular region), GO:0003810 (protein-glutamine gamma-glutamyltransferase activity), GO:0052689 (carboxylic ester hydrolase activity), and GO:0035091 (phosphatidylinositol binding).

## Discussion

We did not find evidence of microgeographic genetic structure among 164 *Ny*. *darlingi* collected from multiple forest cover levels within four Amazonian Brazil municipality groups based on model- and non-model based analyses of genotypes at 5,561 SNP loci. This could indicate that there are high levels of gene flow across populations of *Ny*. *darlingi* in areas with different forest cover levels, consistent with studies in other insects in South America [69, 70] (but see [71]). Our results are not consistent with a previous study in *Ny*. *darlingi* [23], that found microgreographic structure between two municipalities in Brazil with divergent forest cover levels based on analysis of ~2,000 SNP loci generated using ddRADseq. It is possible that the structure between the two municipalities in the Campos *et al*. study was due to unmeasured differences between the two municipalities not related to forest cover, such as differences in breeding site ecology or vector control activities. The current study more comprehensively investigates the relationship between forest cover and population structure, as it includes intermediate forest cover levels, as well as four replicate municipality groups. It is also possible that the adaptive response to forest cover differences varies among *Ny*. *darlingi* populations in different locations due to other factors in the external environment.

Anthropogenic changes in forest cover may produce adaptive phenotypic changes in mosquito populations not reflected in genomic studies, particularly among *Ny*. *darlingi*, as this species has been shown to display a high degree of plasticity in life history traits [72] and biting behavior [44]. Deforestation has been shown to affect the survival and reproductive fitness of *An*. *gambiae* [73], *An*. *arabiensis* [74], and *An*. *minimus* [75]. Additionally, it is possible that whole genome sequencing, or identification of structural variants [76, 77] could identify genetic adaptation to forest cover within *Ny*. *darlingi* populations not reflected in our SNP dataset.

We identified weak to moderate genetic differentiation (*F*_*ST*_ = 0.026–0.075) among four municipality groups across Amazonian Brazil separated by 800–1,600 km. This is consistent with a previous study using microsatellites that found similar *F*_*ST*_ values comparing *Ny*. *darlingi* collected from localities in central and western Amazonian Brazil separated by comparable geographic distances to the current study [34]. However, it contrasts with previous findings that *Ny*. *darlingi* from Amazonian Brazil belong to a single genetic population [33, 35, 36]. This discrepancy could be the result of a combination of increased resolution provided by nextRAD genotyping-by-sequencing [23], different geographic scales of analyses of these studies, and the use of different collection sites. It is clear that more research into the population genetic structure of *Ny*. *darlingi* at both continental and regional scales is needed.

The differentiation that we identified between the municipality groups could be the result of isolation by distance (IBD), isolation by barrier (IBB), isolation by resistance (IBR), or isolation by environment (IBE). It is not possible to differentiate between these possibilities with the sampling scheme of the current study because of the lack of intermediate sampling points between these municipality groups. The overall low level of differentiation between municipality groups, with the highest *F*_*ST*_ (0.075) between the two most geographically separated municipality groups (Presidente Figueiredo and Cruzeiro do Sul area), in combination with a significant Mantel test, is suggestive of IBD. Several previous population genetics studies of *Ny*. *darlingi* using nuclear and mitochondrial markers detected IBD [35, 36, 78, 79], including one study that genotyped *Ny*. *darlingi* at eight microsatellite loci from municipalities in Amazonian Brazil at a similar geographic scale to the current study [34]. However, other studies of *Ny*. *darlingi* have not found evidence of IBD [33, 80, 81]. Additionally, there are geographic barriers between the municipality groups in the current study, including rivers, primary forest, and extensive areas of unsuitable habitats. It is possible that the particular pattern of barriers separating the municipality groups could explain the fact that individuals from SAO and LAB are more similar than other pairs of municipality groups that are separated by a similar distance (Table 3). Future studies could explore the effects of these barriers on genetic divergence between these *Ny*. *darlingi* populations.

We identified 14 SNPs associated with forest cover percentage using two outlier detection methods. These SNPs were located within 100 kb of 150 genes, among which 6 GO terms were over-represented compared with the rest of the *Ny*. *darlingi* genome. We acknowledge that this type of analysis is limited both by the highly fragmented nature of the current *Ny*. *darlingi* genome assembly [45], and by the possibility of false positives [82]. Therefore, we present these results cautiously, with the intention of generating hypotheses for future studies.

### Conclusions

Using nextRAD genotyping-by-sequencing, we report weak genetic structure among four municipality groups in the Amazonian Brazil, but a lack of microgeographic structure across forest cover levels within these municipality groups. These results do not preclude an adaptive response of *Ny*. *darlingi* to deforestation in the Amazon, but indicate that such an adaptive response was not associated with genome-wide differentiation. Additional studies using whole genome sequencing and an improved *Ny*. *darlingi* genome assembly should be undertaken to further explore this topic.

## Supporting information

S1 File Contains Figures A-G and Table A:
• Fig A. Selection of optimal number of clusters for STRUCTURE analysis using the Evanno method.
• Fig B. Results of fastSTRUCTURE analysis of 5561 SNP dataset.
• Fig C. Principal components analysis of 5561 SNP dataset, with shapes and colors indicating the locality and forest cover level of Ny. darlingi collection sites.
• Fig D. Selection of optimal number of clusters for DAPC.
• Fig E. Isolation by distance plot depicting Prevosti’s absolute genetic distance vs. geographic distance in km for pairs of 16 collection sites.
• Fig F. Selection of candidate SNPs using LFMM and bayenv2.
• Fig G. Frequency of major allele for each candidate SNP within each collection site, plotted by the forest cover percentage at each site.
• Table A. GO terms significantly enriched (Fisher’s p<0.01) within 100kb of candidate SNPs in Ny. darlingi genome.
(PDF)

S2 File Sample information table, including collection information, coordinates, sequencing and STACKS data, and SRA accession number for 190 sequenced *Ny. darlingi*. (XLSX)

S3 File Collection details of 24 *Ny. darlingi* used to build STACKS catalog, including collection date, location, and associated publication. (XLSX)

S4 File Bash script including all Stacks commands used to build loci and call SNPs from HiSeq reads. (TXT)

S5 File Structure file of 5561 SNPs genotyped in 164 individual *Ny. darlingi* used for all analyses. (STR)

S6 File Annotation of 150 genes in *Ny. darlingi* genome located within 100 kb of 14 candidate SNPs, including GO terms, OrthoDB identifiers for Diptera level orthology, *D. melanogaster* gene names and FlyBase ID numbers were available. (CSV)
