# Supplementary material for: Minimal genetic differentiation of the malaria vector Nyssorhynchus darlingi associated with forest cover level in Amazonian Brazil
Source: PLoS One. 2019 Nov 14;14(11):e0225005. doi: 10.1371/journal.pone.0225005 (PMC6855485; doi:10.1371/journal.pone.0225005)
Supplement: S1 File — Contains Figures A-G and Table A: • Fig A. Selection of optimal number of clusters for STRUCTURE analysis using the Evanno method. • Fig B. Results of fastSTRUCTURE analysis of 5561 SNP dataset. • Fig C. Principal components analysis of 5561 SNP dataset, with shapes and colors indicating the locality and forest cover level of Ny. darlingi collection sites. • Fig D. Selection of optimal number of clusters for DAPC. • Fig E. Isolation by distance plot depicting Prevosti’s absolute genetic distance vs. geographic distance in km for pairs of 16 collection sites. • Fig F. Selection of candidate SNPs using LFMM and bayenv2. • Fig G. Frequency of major allele for each candidate SNP within each collection site, plotted by the forest cover percentage at each site. • Table A. GO terms significantly enriched (Fisher’s p<0.01) within 100kb of candidate SNPs in Ny. darlingi genome. (PDF) [file pone.0225005.s001.pdf]

S1 File: Supplementary Figures and Tables

Minimal genetic differentiation of the malaria vector *Nyssorhynchus darlingi* associated with forest cover level in Amazonian Brazil

Catharine Prussing, Kevin J. Emerson, Sara A. Bickersmith, Maria Anice Mureb Sallum, and Jan E. Conn

Fig A. Selection of optimal number of clusters for STRUCTURE analysis using the Evanno method. (A) Posterior probability of the data (L(K)) for *K*=1 to 10, (B) L'(K) for *K*=1 to 9, and (C) L''(K) and (D) delta K for *K*=2 to 9.

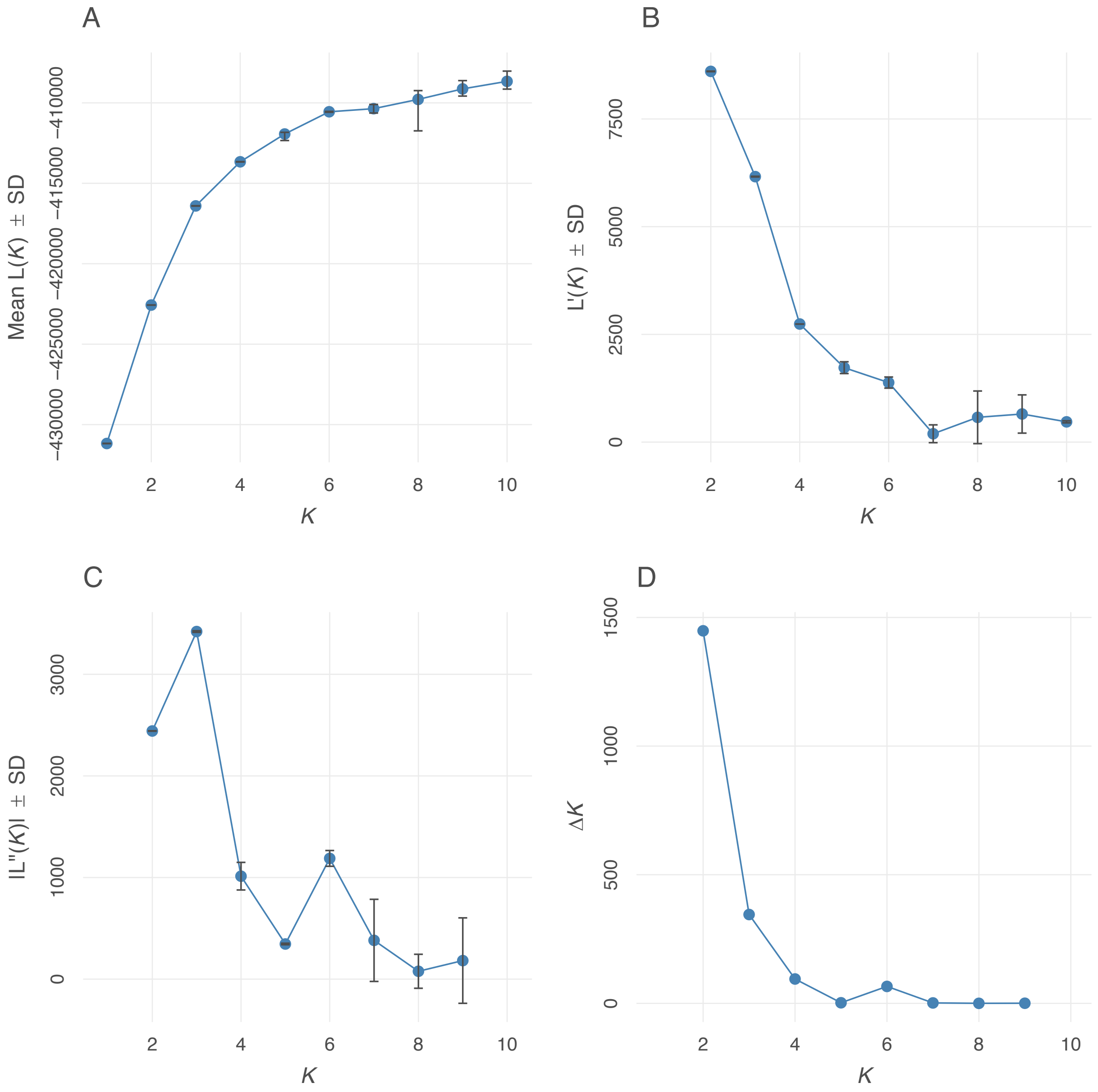

Fig B. Results of fastSTRUCTURE analysis of 5561 SNP dataset. (A) Includes *Ny. darlingi* collected from all four municipalities (CRU: Cruzeiro do Sul area, LAB: Lábrea, PRE: Presidente Figueiredo, SAO: São Gabriel da Cachoeira) and five forest cover levels (1-5), depicting *K*=2-6 inferred clusters; (B) CRU only, *K*=1-5; (C) LAB only, *K*=1-5; (D) PRE only, *K*=1-5; (E) SAO only, *K*=1-5.

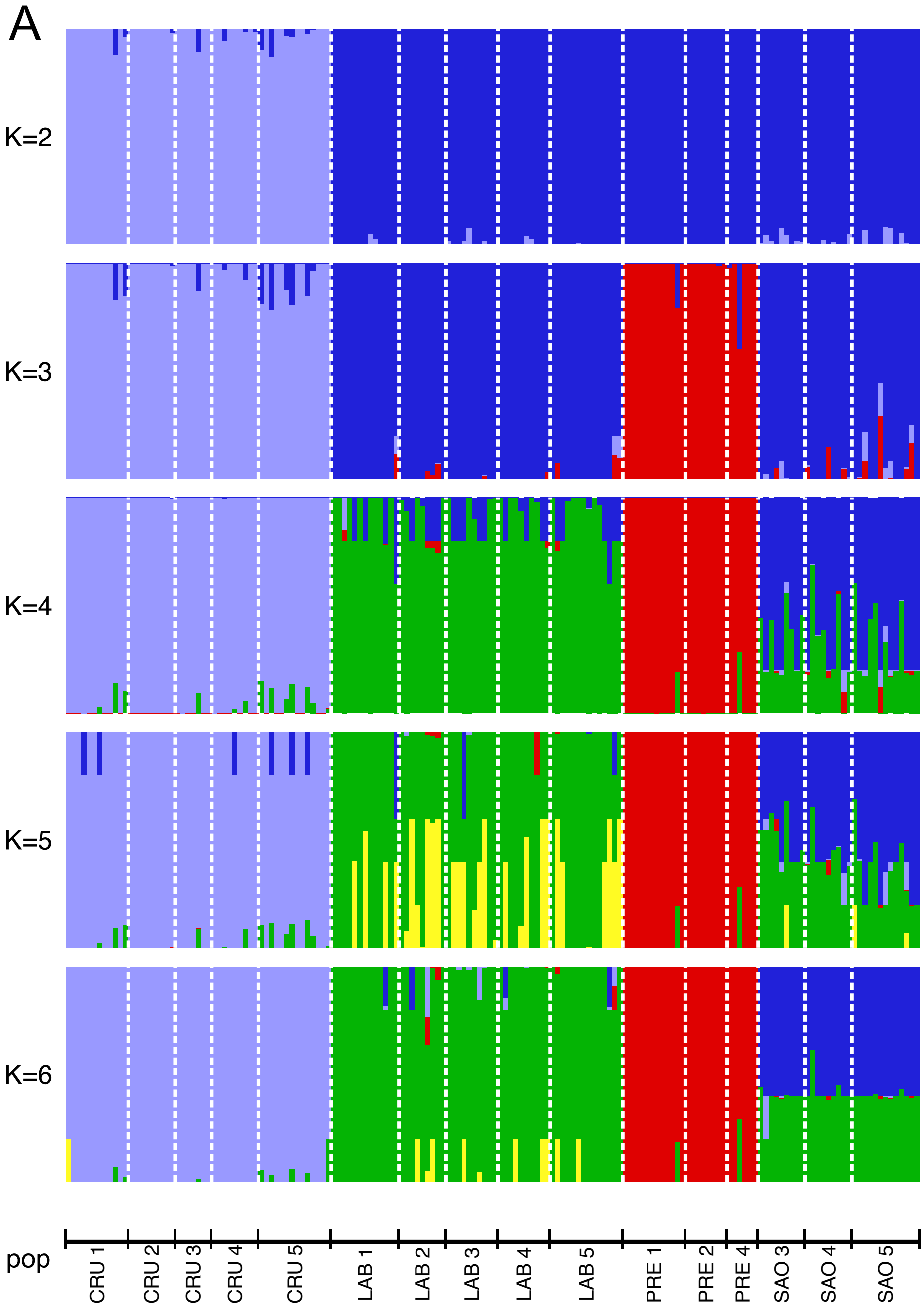

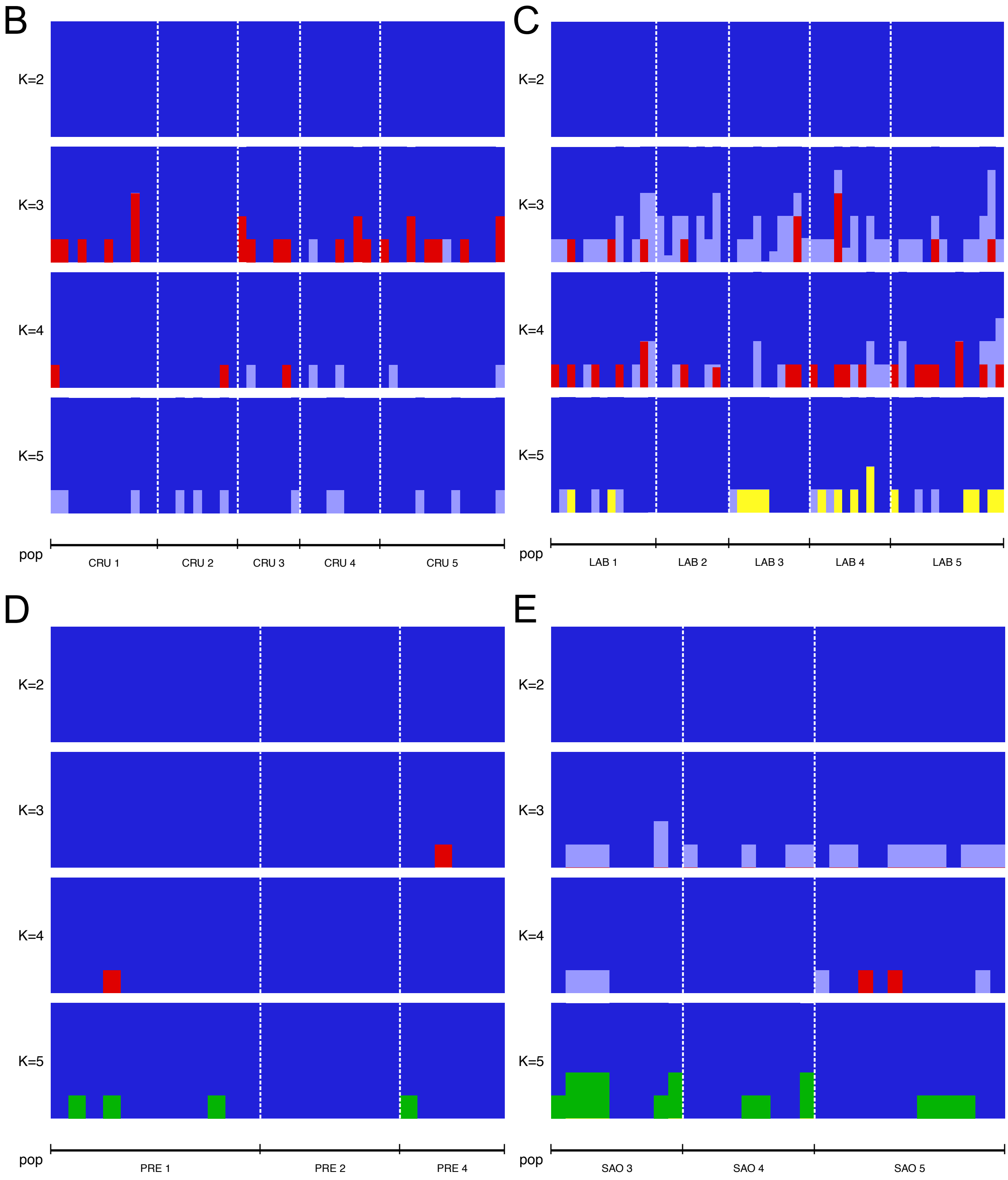
B
K=2
K=3
K=4
K=5
pop
CRU 1
CRU 2
CRU 3
CRU 4
CRU 5
C
K=2
K=3
K=4
K=5
pop
LAB 1
LAB 2
LAB 3
LAB 4
LAB 5
D
K=2
K=3
K=4
K=5
pop
PRE 1
PRE 2
PRE 4
E
K=2
K=3
K=4
K=5
pop
SAO 3
SAO 4
SAO 5

Fig C. Principal components analysis of 5561 SNP dataset, with shapes and colors indicating the locality and forest cover level of *Ny. darlingi* collection sites.

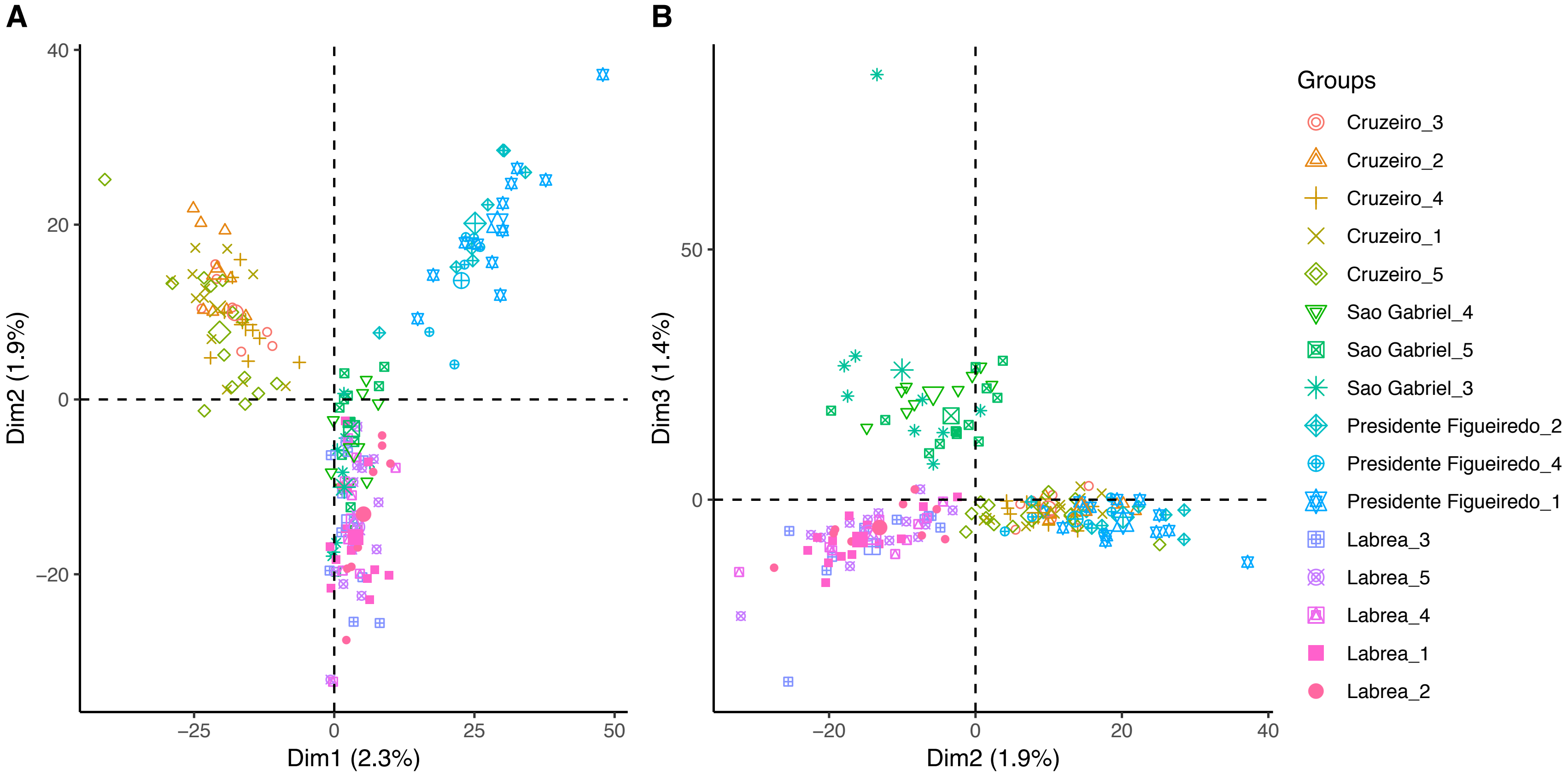

Fig D. Selection of optimal number of clusters for Discriminant Analysis of Principal Components (DAPC). (A) Bayesian Information Criterion (BIC) and (B) Akaike Information Criterion (AIC) for 1 to 30 clusters using K-means clustering in preparation for DAPC.

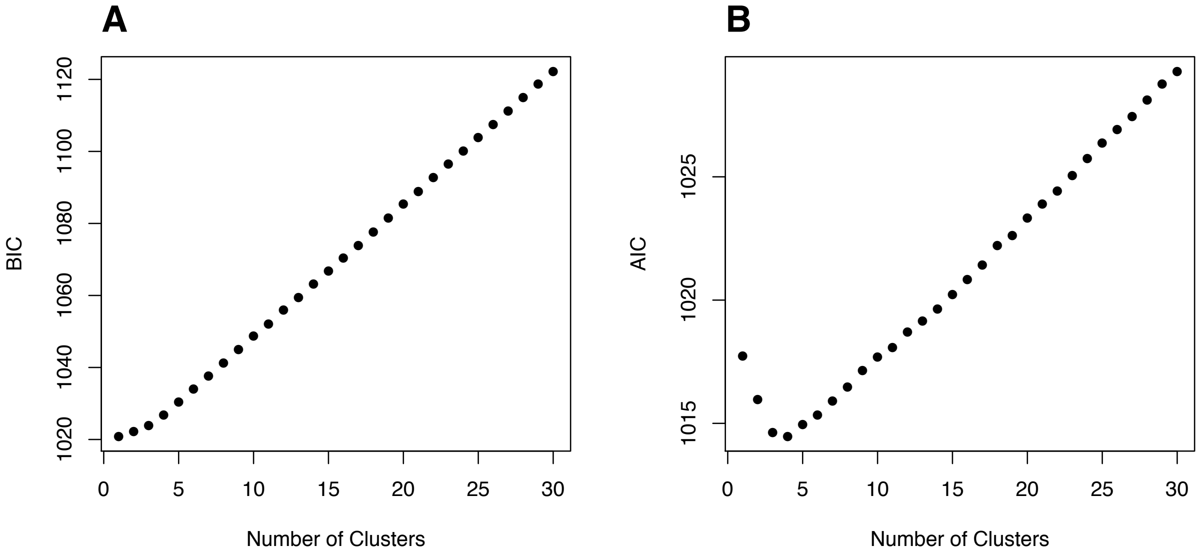

Fig E. Isolation by distance plot depicting Prevosti's absolute genetic distance vs. geographic distance in km for pairs of 16 collection sites. Each dot represents a pairwise comparison between two collection sites.

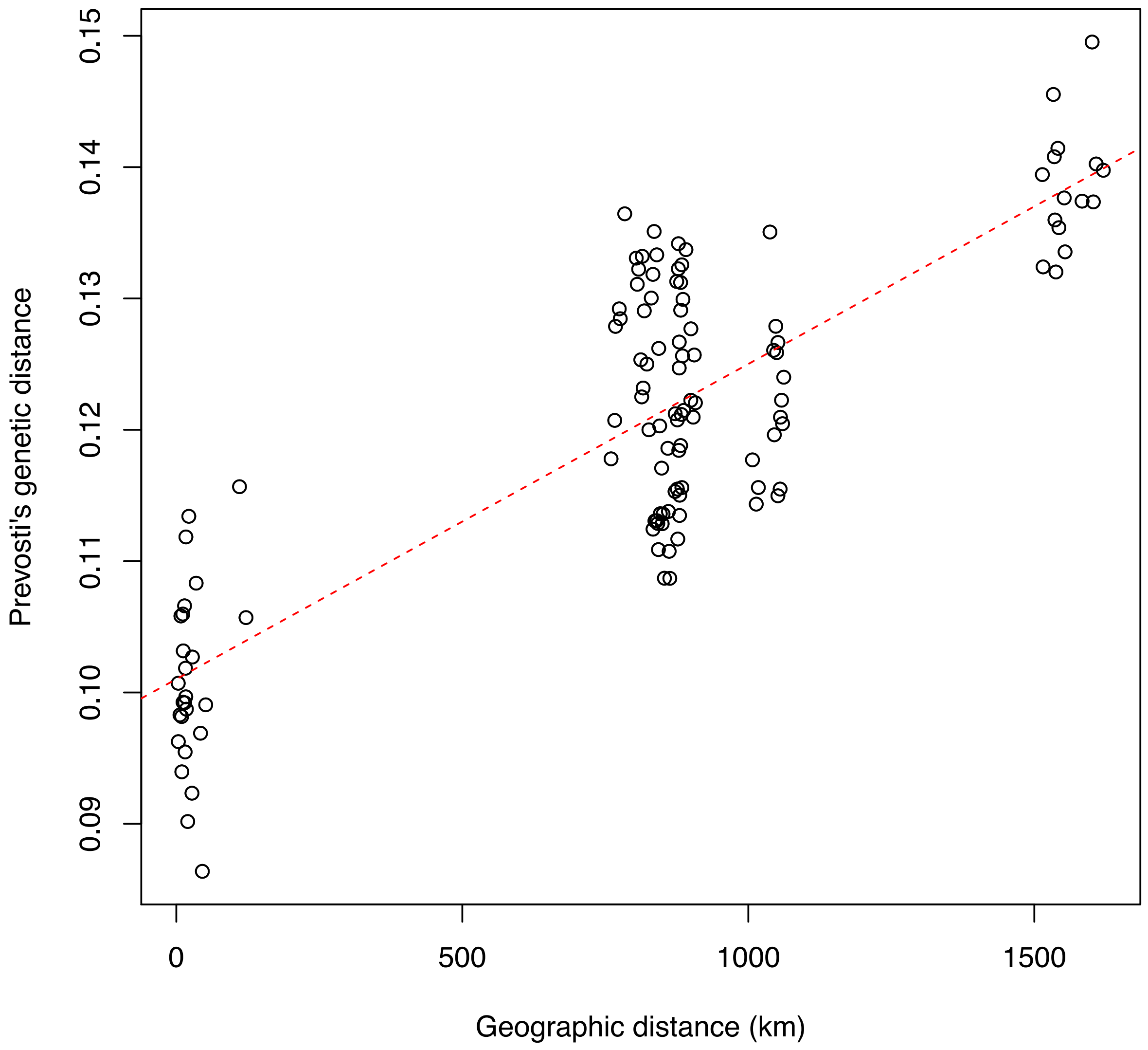

Fig F. Selection of candidate SNPs. (A) Plot of negative log of adjusted LFMM *p*-values for 5561 SNPs, highlighting SNPs with *p*<0.05. (B) Plot of log of bayenv2 Bayes factors and absolute value of Spearman's *ρ* for 5561 SNPs, highlighting SNPs in the top 5% of Bayes factors and the top 10% of Spearman's *ρ*.

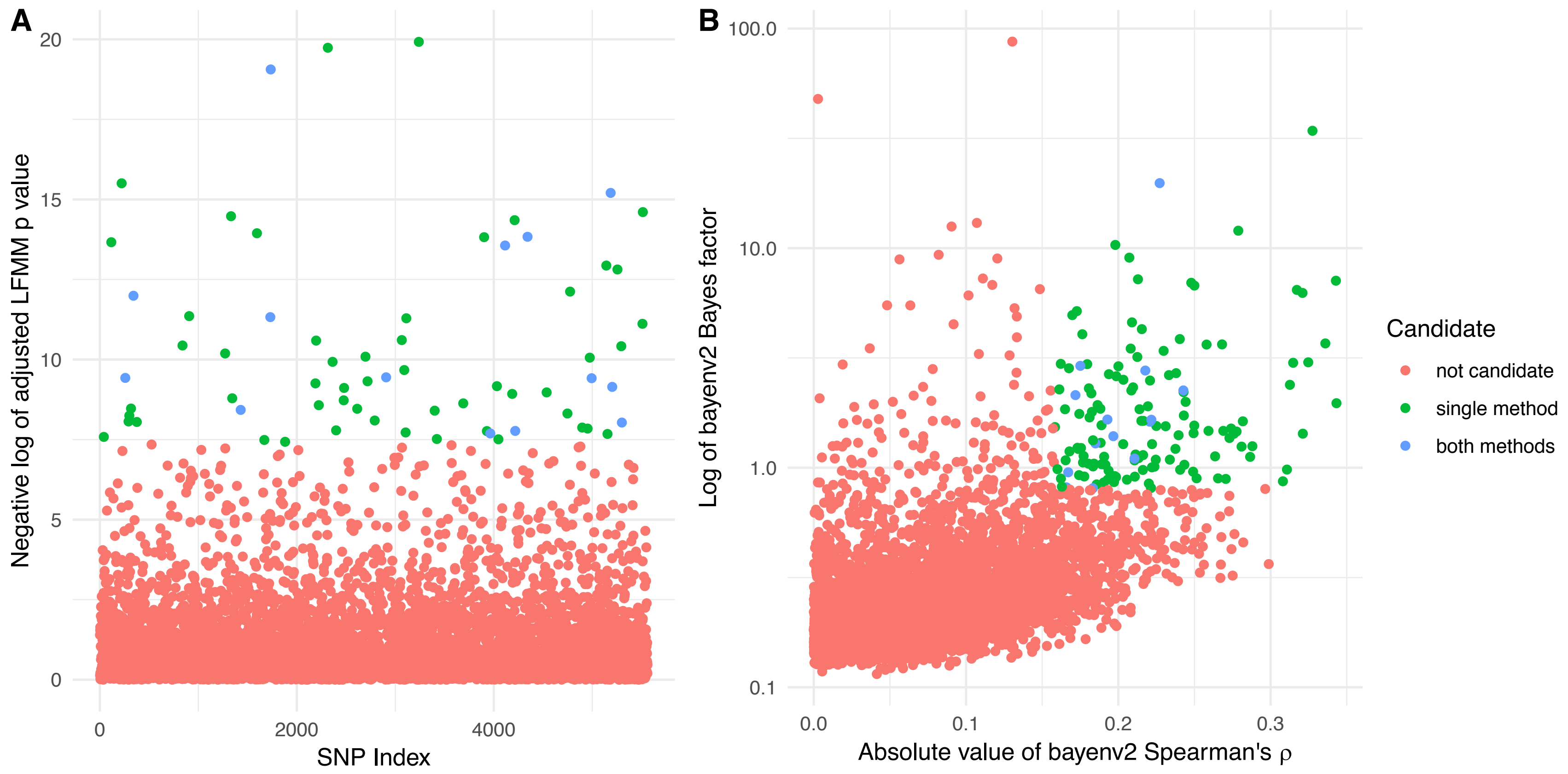

Fig G. Frequency of major allele for each candidate SNP within each collection site, plotted by the forest cover percentage at each site.

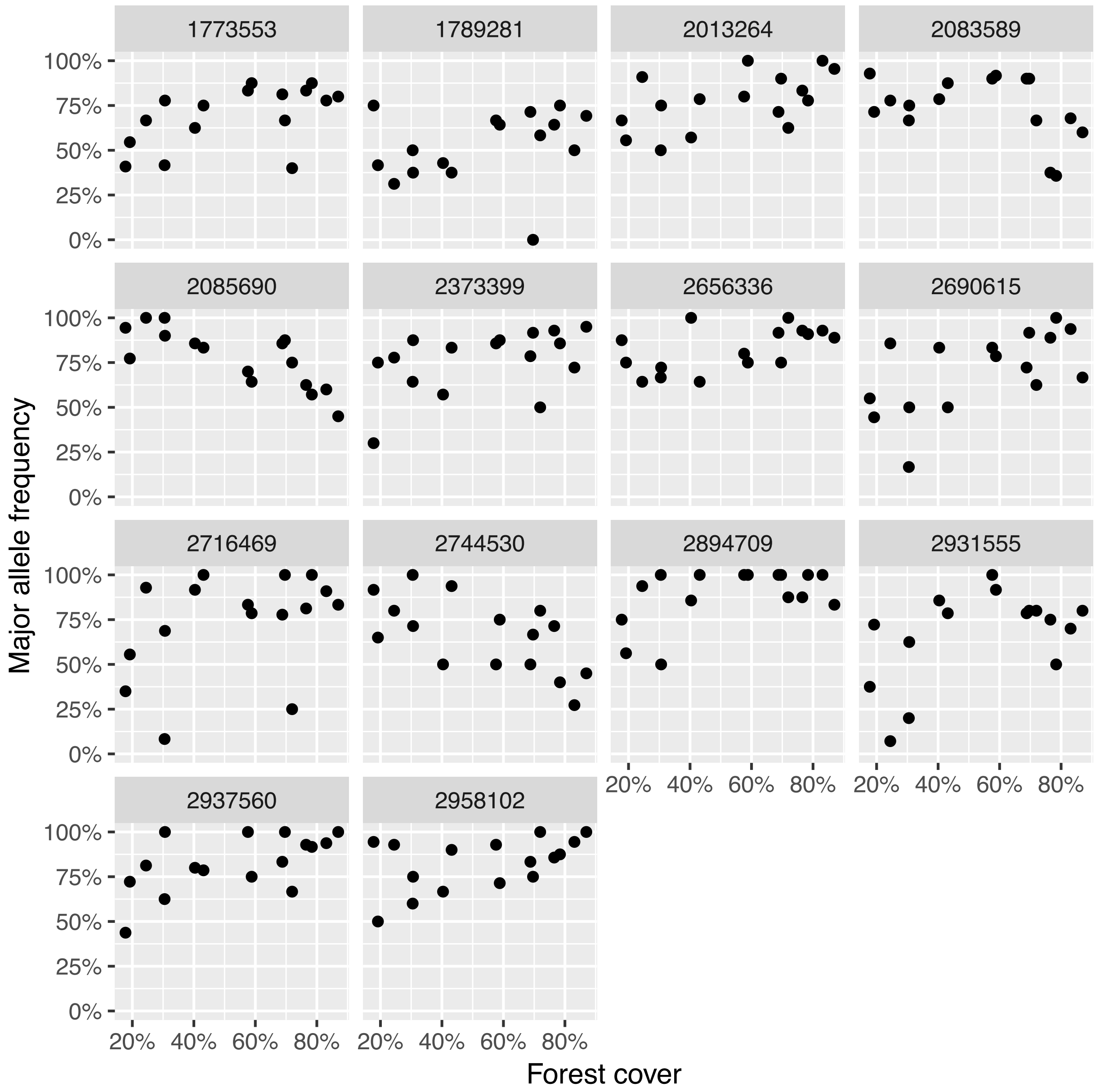

Table A. GO terms significantly enriched (Fisher's *p*<0.01) within 100kb of candidate SNPs in *Ny. darlingi* genome.

GO Sub-ontology	GO ID	Description	Number in annotated genome	Number within 100kb of candidate SNPs	Expected	Fisher's *p*-value
Biological Process (BP)	GO:0035278	miRNA mediated inhibition of translation	3	2	0.04	0.0005
Biological Process (BP)	GO:0018149	peptide cross-linking	3	2	0.04	0.0005
Cellular Component (CC)	GO:0005576	extracellular region	178	8	1.82	0.0004
Molecular Function (MF)	GO:0003810	protein-glutamine gamma-glutamyltransferase activity	3	2	0.04	0.0006
Molecular Function (MF)	GO:0052689	carboxylic ester hydrolase activity	51	4	0.75	0.007
Molecular Function (MF)	GO:0035091	phosphatidylinositol binding	27	3	0.4	0.007
